# Supplementary figures and images for: Global reduction of in situ CO2 transfer velocity by natural surfactants in the sea-surface microlayer
Source: Proc Math Phys Eng Sci. 2020 Feb 12;476(2234):20190763. doi: 10.1098/rspa.2019.0763 (PMC7069489; doi:10.1098/rspa.2019.0763)

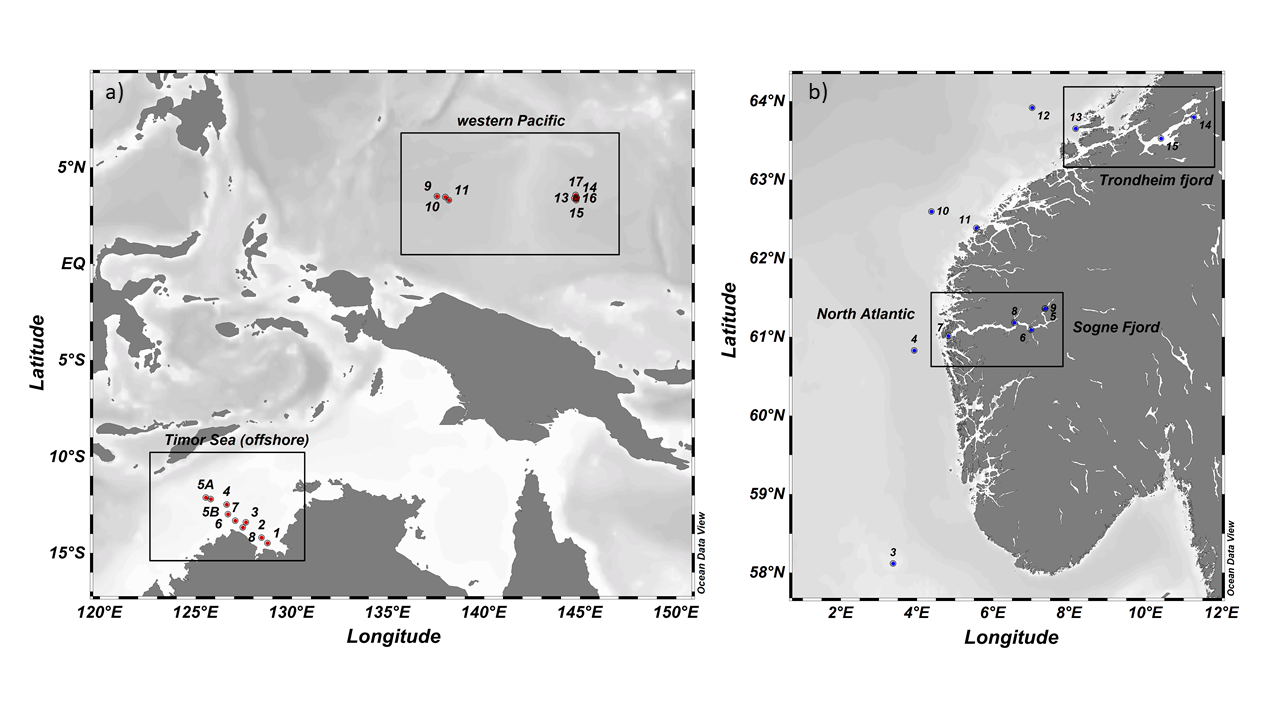

Supplement: Figure S1. Map of study areas. [file rspa20190763supp1.tif]

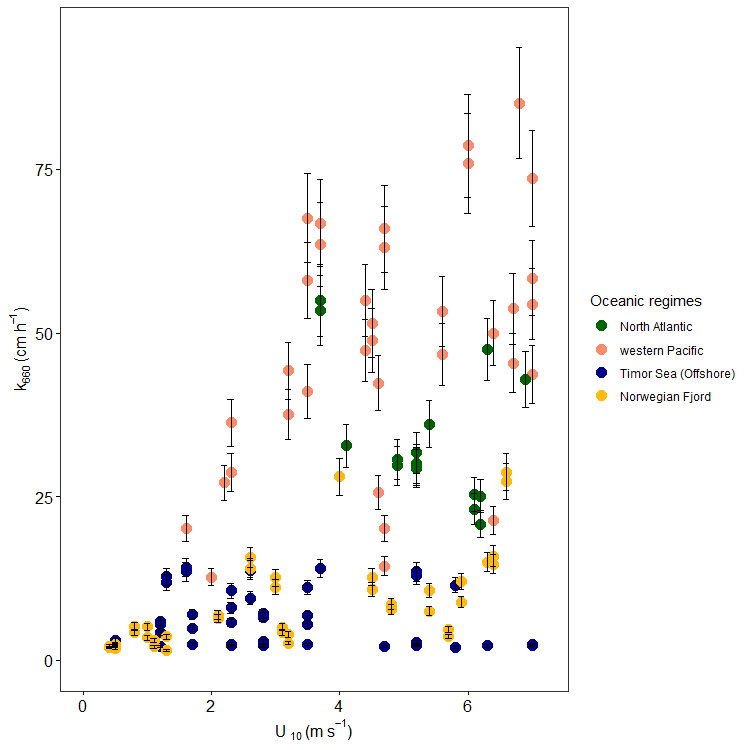

Supplement: Figure S2. Scattered plots of in-situ k660 and wind speed (U10). [file rspa20190763supp2.tiff]

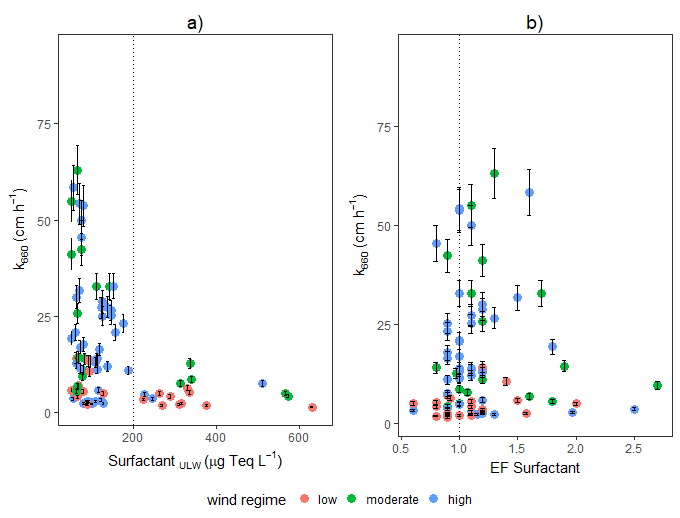

Supplement: Figure S3. Scattered plots of k660 and surfactant. [file rspa20190763supp3.tiff]
