# Supplementary material for: Global reduction of in situ CO2 transfer velocity by natural surfactants in the sea-surface microlayer
Source: Proc Math Phys Eng Sci. 2020 Feb 12;476(2234):20190763. doi: 10.1098/rspa.2019.0763 (PMC7069489; doi:10.1098/rspa.2019.0763)
Supplement: Table S1. Estimation of air-sea CO2 fluxes reduction by surfactants in the western Pacific, North Atlantic and Norwegian Fjords. [file rspa20190763supp4.docx]

**Table S1.** Estimation of air-sea CO_2_ fluxes reduction by surfactants in the western Pacific, North Atlantic and Norwegian Fjords.

|  | ① | ② | ③ | ④ | Reduction of CO_2_ fluxes by surfactants^c^ | |
| --- | --- | --- | --- | --- | --- | --- |
|  | Area (km^2^) | Fraction (%) | CO_2_ fluxes | % coverage by non-slicks |  |  |
|  |  |  | (Tg C year^-1^) |  | ⑤ Tg C year^-1^ | ⑥ % |
| Pacific ocean^a^ | 153.8 x 10^6^ | 100 | -0.46^a^ | 89 | -0.09 | 20 |
| western Pacific | 3.7 x 10^6^ | 2.4 | -0.01 | 89 | -2.3 x 10^-3^ | 20 |
| Atlantic ocean^a^ | 74.6 x 10^6^ | 100 | -0.58^a^ | 89 | -0.12 | 20 |
| North Atlantic | 0.14 x 10^6^ | 0.2 | -0.001 | 89 | -2.4 x 10^-4^ | 20 |
| Norwegian Fjords | 1183 | 0.0016 | -9.19 x 10^-6^ | 70 | -2.0 x 10^-6^ | 16 |
|  |  |  | ③ = CO_2_ fluxes^a^ *②/100 | ④ = 100 - % slicks coverage^b^ | ⑤ = ③ *(④/100) *0.23^c^ | ⑥ = ⑤*100/③ |

^a^ Based on estimated CO_2_ fluxes (Tg C year^-1^) (*57*)

^b^ Frequency of ocean coverages by non-slicks by considering slicks coverages in the coastal (30%) and open ocean (11%) (*6*)

^c^ Based on 23% reduction by surfactants from our study
