# Supplementary material for: Global reduction of in situ CO2 transfer velocity by natural surfactants in the sea-surface microlayer
Source: Proc Math Phys Eng Sci. 2020 Feb 12;476(2234):20190763. doi: 10.1098/rspa.2019.0763 (PMC7069489; doi:10.1098/rspa.2019.0763)
Supplement: Table S2. Estimation of air-sea CO2 fluxes reduction by slicks in the western Pacific, North Atlantic and Norwegian Fjords. [file rspa20190763supp5.docx]

**Table S2.** Estimation of air-sea CO_2_ fluxes reduction by slicks in the western Pacific, North Atlantic and Norwegian Fjords.

|  | ① | ② | ③ | ④ | Reduction of CO_2_ fluxes by slicks^c^ | |
| --- | --- | --- | --- | --- | --- | --- |
|  | Area (km^2^) | Fraction (%) | CO_2_ fluxes | % coverage by slicks^b^ |  |  |
|  |  |  | (Tg C year^-1^) |  | ⑤ Tg C year^-1^ | ⑥ % |
| Pacific ocean^a^ | 153.8 x 10^6^ | 100 | -0.46^a^ | 11 | -0.03 | 7 |
| western Pacific | 3.7 x 10^6^ | 2.4 | -0.01 | 11 | -7.55 x 10^-4^ | 7 |
| Atlantic ocean^a^ | 74.6 x 10^6^ | 100 | -0.58^a^ | 11 | -3.95 x 10^-2^ | 7 |
| North Atlantic | 0.14 x 10^6^ | 0.2 | -0.001 | 11 | -7.33 x 10^-5^ | 7 |
| Norwegian Fjords | 1183 | 0.0016 | -9.19 x 10^-6^ | 30 | -1.71 x 10^-6^ | 19 |
|  |  |  | ③ = CO_2_ fluxes^a^ *②/100 |  | ⑤ = ③ *(④/100) *0.62^c^ | ⑥ = ⑤*100/③ |

^a^ Based on estimated CO_2_ fluxes (Tg C year^-1^) (*57*)

^b^ Frequency of ocean coverages by slicks in the coastal (30%) and open ocean (11%) (*6*)

^c^ Based on 62% reduction by slicks from our study
